# Gestational progesterone restores menstrual cycle in PCOS patients via enhancing ovary estrogen production

**DOI:** 10.1093/lifemeta/loag004

**Published:** 2026-02-02

**Authors:** Qiwen Yang, Na Kong, Jing Wu, Yan Yang, Xinge Zhang, Yuan Lin, Zhibin Hu, Guijun Yan, Haixiang Sun, Chaojun Li

**Affiliations:** State Key Laboratory of Reproductive Medicine and Offspring Health, Nanjing Medical University, Nanjing, Jiangsu 211166, China; Center for Reproductive Medicine and Obstetrics & Gynecology, Nanjing Drum Tower Hospital, Affiliated Hospital of Medical School, Nanjing University, Nanjing, Jiangsu 210008, China; Center for Reproductive Medicine and Obstetrics & Gynecology, Nanjing Drum Tower Hospital, Affiliated Hospital of Medical School, Nanjing University, Nanjing, Jiangsu 210008, China; State Key Laboratory of Reproductive Medicine and Offspring Health, Nanjing Medical University, Nanjing, Jiangsu 211166, China; State Key Laboratory of Reproductive Medicine and Offspring Health, Nanjing Medical University, Nanjing, Jiangsu 211166, China; State Key Laboratory of Reproductive Medicine and Offspring Health, Nanjing Medical University, Nanjing, Jiangsu 211166, China; State Key Laboratory of Reproductive Medicine and Offspring Health, Nanjing Medical University, Nanjing, Jiangsu 211166, China; Center for Reproductive Medicine and Obstetrics & Gynecology, Nanjing Drum Tower Hospital, Affiliated Hospital of Medical School, Nanjing University, Nanjing, Jiangsu 210008, China; Center for Reproductive Medicine and Obstetrics & Gynecology, Nanjing Drum Tower Hospital, Affiliated Hospital of Medical School, Nanjing University, Nanjing, Jiangsu 210008, China; State Key Laboratory of Reproductive Medicine and Offspring Health, Nanjing Medical University, Nanjing, Jiangsu 211166, China; Changzhou Maternity and Child Health Care Hospital, Changzhou Medical Center, Nanjing Medical University, Changzhou, Jiangsu 213000, China

**Keywords:** polycystic ovary syndrome, menstrual cycle, progesterone, granulosa cell, apoptosis

## Abstract

Polycystic ovary syndrome (PCOS) is the most common endocrine disorder among women of reproductive age, typically characterized by irregular menstrual cycles. Our study found that postpartum menstrual cycles were largely restored in PCOS patients following assisted reproductive technology (ART) therapy. However, this recovery in menstrual cycles was not ­associated with any specific ART procedures. Using a PCOS mouse model, we demonstrated that elevated progesterone levels during pregnancy were responsible for normalizing estrous cyclicity. Elevated levels of progesterone induced granulosa cell apoptosis and depleted large follicles, which potentially contributed to ovarian function suppression during pregnancy. Mechanistic studies indicated that progesterone decreased follicle-stimulating hormone receptor (FSHR) expression in a GATA binding protein 2 (GATA2)-dependent manner. Interestingly, the capacity of granulosa cells to convert androgens to estrogens significantly increased after progesterone withdrawal, as evidenced by elevated expression of cytochrome P450 family 19 subfamily A member 1 (Cyp19a1) in granulosa cells when stimulated with follicle-stimulating hormone. In addition, we found that progesterone administration reduced the thickness of the uterine endometrium in PCOS mice. Our findings suggest that sustained high levels of progesterone during pregnancy can enhance ovarian reproductive endocrine capacity and improve endometrial function, thereby facilitating the recovery of postpartum menstrual cycles.

## Introduction

Polycystic ovary syndrome (PCOS) is the primary cause of anovulatory subfertility, and affects 6%–20% of women of reproductive age worldwide [1, 2]. It is characterized by a diverse range of clinical symptoms, including hyperandrogenism, menstrual cycle irregularities (amenorrhea or oligomenorrhea), ovulatory dysfunction, polycystic ovarian morphology, and often, metabolic abnormalities [3, 4]. In women with PCOS, the pulse frequency of hypothalamic gonadotropin-releasing hormone (GnRH) is elevated, resulting in an altered luteinizing hormone: follicle-stimulating hormone (LH:FSH) ratio. This disruption leaves the ovaries in a persistent early follicular state, depriving them of essential endocrine signals required for ovulation. Excessive LH stimulates androgen synthesis in follicular theca cells, leading to increased ovarian androgen production. Elevated androgen levels, in turn, inhibit the function of ovarian granulosa cells and disrupt the expression of follicle-stimulating hormone receptor (FSHR), potentially reducing their responsiveness to FSH. This process can cause activated follicles to be arrested before maturation, resulting in ovulatory dysfunction and polycystic ovarian morphology [5–7]. Ultimately, this aberrant hormone production and signaling lead to asynchronous endometrial proliferation, causing ­irregular and unpredictable endometrial shedding, which is the ­primary cause of menstrual cycle disorders in PCOS patients [8, 9]. Currently, however, no effective methods exist to alleviate or treat menstrual cycle irregularities associated with PCOS.

During pregnancy, endocrine hormones—particularly progesterone and estrogen—regulate the adaptive changes in the reproductive system to meet the needs of the developing fetus. Generally, steroid hormone levels increase significantly throughout gestation to support the physiological demands necessary for a successful pregnancy. In early pregnancy, progesterone is mainly produced by ovarian luteal cells; however, the placenta becomes the predominant source of steroid hormones for the ­duration of the pregnancy. Initially, progesterone levels are ­approximately 13.00 ± 1.65 ng/mL during the first trimester, increasing to 15.81 ± 1.03 ng/mL in the second trimester. By the third trimester, progesterone levels can reach as high as 89.93 ± 11.99 ng/mL [10,  11]. Following childbirth, the levels of progesterone and estrogen decrease, alleviating their inhibitory effects on the hypothalamus and pituitary gland. Consequently, the hypothalamus resumes GnRH secretion, leading to increased FSH secretion from the pituitary [12]. The significant hormonal changes occurring during and after pregnancy prompt us to speculate whether these drastic fluctuations can affect the status of neuroendocrine disorders such as PCOS.

This study aimed to clarify the specific effects of increased progesterone levels during pregnancy in patients with PCOS. Our findings demonstrate that postpartum menstrual cycles were significantly improved in these patients. Utilizing a PCOS-like mouse model, we demonstrated that sustained high levels of progesterone during pregnancy promoted the recovery of menstrual cycles in PCOS patients. This hormonal environment may enhance ovarian endocrine function and improve endometrial function after pregnancy.

## Results

### The postpartum menstrual cycle shows improvement in patients with PCOS following delivery

**Figure 1:**
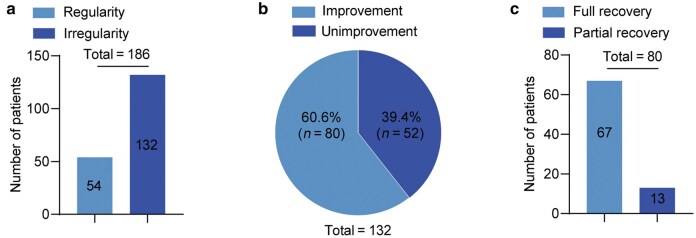
Improvements of menstrual cycle conditions in PCOS patients following ART-conceived pregnancy. (a) All patients are categorized into two groups: the group with regular menstrual cycles (54 patients) and the group with irregular menstrual cycles (132 patients). (b) Patients with irregular menstrual cycles are divided into the unimprovement group (52 patients) and the improvement group (80 patients). (c) Improvement conditions in patients with irregular menstrual cycles.

We analyzed the menstrual cycles of a total of 186 patients with PCOS who successfully delivered following assisted reproductive technology (ART). Out of 132 PCOS patients with irregular cycles (Fig. 1a), 80 patients experienced improvements in their menstrual cycles after delivery (60.60%; Fig. 1b), with 67 fully recovering to normal menstrual patterns and 13 partially improving (Fig. 1c). The results presented in Table 1 indicate that there were no significant differences between patients with and without improvements in age, years of infertility, treatment duration, body mass index (BMI), baseline sex hormones, or basal antral follicles. As shown in Table 2, there were also no significant differences between patients in the two groups regarding the ovulation induction process or the outcomes of ART and pregnancy. This included parameters such as downregulating dosage, number of oocytes harvested, number of available embryos, type of transplanted embryos, incidence of ovarian hyperstimulation syndrome, mode of delivery, and ­gestational age.

**Table 1:** Baseline clinical characteristics.

	Group A (*n *= 52)	Group B (*n *= 80)	*P* value
**Age (year)**	28.2 ± 3.3	28.1 ± 3.6	0.921
**Duration of infertility**	3.0 (0.5–12.0)	3.0 (1.0–11.0)	0.320
**Total treatment period**	1.4 ± 0.9	1.2 ± 0.7	0.112
**BMI (kg/m^2^)**	23.2 ± 3.1	24.0 ± 3.8	0.279
**FSH (mIU/mL)**			
**Basic**	6.3 ± 1.8	6.3 ± 1.7	0.452
**Start-up date**	3.0 ± 1.5	3.3 ± 1.4	0.226
**Trigger date**	12.6 ± 4.3	12.5 ± 3.9	0.514
**LH (mIU/mL)**			
**Basic**	8.9 ± 4.6	7.7 ± 5.4	0.151
**Start-up date**	0.8 ± 1.1	0.6 ± 0.4	0.797
**Trigger date ** **E2 (pg/mL)**	1.0 ± 0.6	0.9 ± 0.5	0.399
**Basic**	49.2 ± 40.2	44.0 ± 36.4	0.581
**Start-up date**	16.3 ± 10.7	20.3 ± 19.3	0.342
**Trigger date ** **P (ng/μL)**	3,654.4 ± 1399.1	3,409.1 ± 1,267.8	0.286
**Start-up date**	0.3 ± 0.8	0.1 ± 0.2	0.312
**Trigger date**	0.8 ± 0.5	0.6 ± 0.4	0.241
**T (ng/μL)**	0.7 ± 0.3	0.6 ± 0.3	0.147
**PRL**	13.25	13.46	0.64
**Basal antral follicles**	22.4 ± 2.9	22.3 ± 3.8	0.754

**Table 2:** Ovulation induction process and pregnancy outcome.

	Group A (*n *= 52)	Group B (*n *= 80)	*P* value
**The falling tone dose**	2.8 ± 1.1	2.5 ± 1.1	0.229
**Numbers of retrieved oocytes**	13.1 ± 3.2	12.2 ± 3.4	0.192
**Number of mature oocytes**	11.6 ± 3.6	11.4 ± 3.6	0.684
**Normal fertilization number**	9.2 ± 3.7	8.7 ± 3.1	0.754
**Number of available embryos**	4.8 ± 2.2	4.6 ± 2.0	0.853
**Average number of embryos transferred**	1.9 ± 0.2	1.9 ± 0.2	0.868
**Embryo type *n* (%)**			1.000
**Cleavage embryo**	50 (96.2%)	78 (97.4%)	
**One blastocyst**	2 (3.8%)	2 (2.6%)	
**Endometrial thickness before transplantation (mm)**	11.3 ± 3.6	11.7 ± 1.9	0.600
**Incidence of ovarian hyperstimulation**	14(27%)	13(16.9%)	0.381
**Embryo implantation rate**	76.24%	78.52%	0.607
**Delivery gestational age**	37.3 ± 2.4	36.9 ± 3.2	0.833
**Delivery pattern**			0.714
**Eutocia**	25%	24.4%	
**Caesarean section**	75%	75.6%	
**Duration of breast feeding (month)**	7.8 ± 5.9	8.9 ± 6.0	0.359
**Postpartum BMI change**	1.5 ± 3.1	0.3 ± 2.2	0.020*
**Decline**	7(16.7%)	26 (41.3%)	
**Increase**	35 (83.3%)	37 (58.7%)	

The results suggest that the improvements in menstrual cycles among PCOS patients were not influenced by their clinical characteristics or any ART procedures. Totally, 60.60% of PCOS patients experienced improvements in their menstrual cycles following delivery. However, the specific mechanisms underlying this ­improvement remain to be elucidated.

### Administering progesterone restores normal estrous cyclicity in PCOS mice

The regulatory process of the menstrual cycle is complex, involving intricate interactions among the hypothalamus, pituitary gland, ovaries, and uterus. We hypothesized that the improvements of menstrual cycles in PCOS patients after delivery may stem from multiple factors, including psychological benefits associated with normal delivery, inflammation and immune modifications related to childbirth, and hormonal fluctuations during pregnancy, delivery, and lactation. To investigate the underlying mechanisms, we established a PCOS-like model in mice by administering letrozole, an aromatase inhibitor, for 3 weeks starting at postnatal day 21. Letrozole-treated (Let group) mice exhibited major criteria for PCOS diagnosis in humans, including increased ovary index (Supplementary Fig. S1a–c; see online supplementary material for a color version of this figure), hyperandrogenism (Supplementary Fig. S1d), elevated LH levels (Supplementary Fig. S1e; see online supplementary material for a color version of this figure), prolonged anogenital distance (Supplementary Fig. S1f; see online supplementary material for a color version of this figure), and abnormal estrous cyclicity (Supplementary Fig. S1g and h; see online supplementary material for a color version of this figure). Furthermore, glucose tolerance and insulin sensitivity were lower in the Let group compared to the controls (Supplementary Fig. S1i and j; see online supplementary material for a color version of this figure).

**Figure 2:**
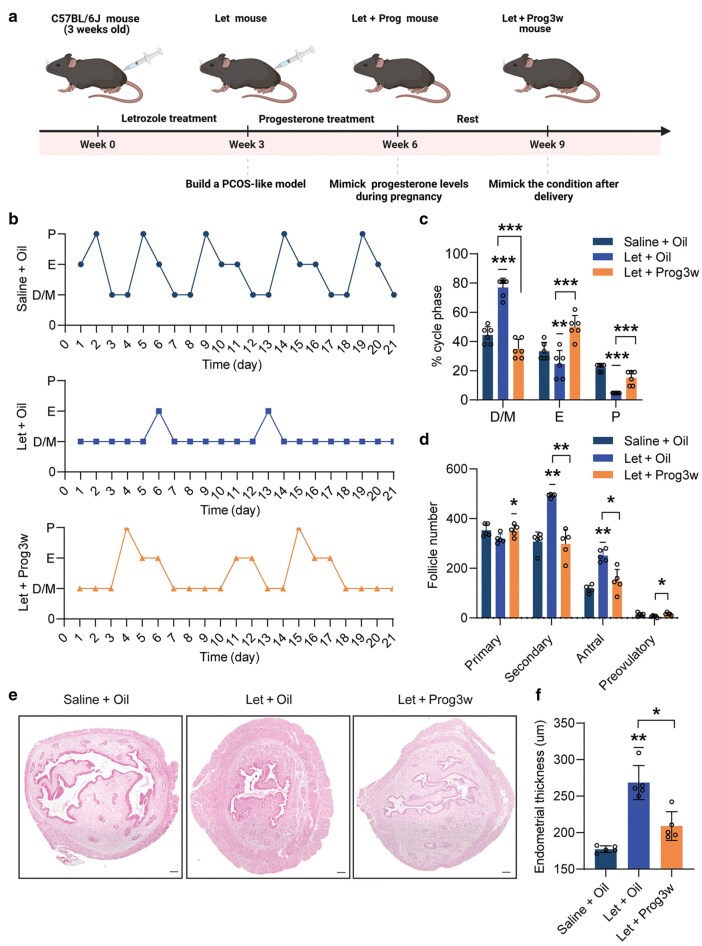
Progesterone treatment improves endocrine reproductive phenotypes in a PCOS-like mouse model. (a) Flowchart of PCOS mouse modeling and progesterone treatment. (b) Representative estrous cyclicity observed in mice of control, Let, and Prog3w groups over 21 consecutive days (*n *= 6). M/D: metestrus/diestrus phase; P: proestrus; E: estrus. (c) Quantitative analysis of estrous cyclicity in mice of control, Let, and Prog3w groups, displayed as a scatterplot representing the percentage (%) of time spent in each estrous phase (*n *= 6). (d) Quantitative assessment of follicle counts at different developmental stages in mice of control, Let, and Prog3w groups (*n *= 5). (e) Hematoxylin and eosin staining of representative uterus in mice of control, Let, and Prog3w groups. Scale bar: 100 μm. (f) Statistical analysis of average endometrial thickness (*n *= 5).

We then treated the PCOS mice with high levels of progesterone for 3 weeks (Prog3w group) to mimic the elevated progesterone levels during pregnancy (Fig. 2a), followed by an examination of their estrous cyclicity after progesterone withdrawal. The PCOS mice exhibited abnormal estrous cyclicity characterized by prolonged diestrus and metestrus phases, along with sparse estrus and post-estrus (Fig. 2b and c). However, progesterone treatment effectively improved estrous cyclicity, restoring both estrus and post-estrus phases (Fig. 2b and c). In addition, progesterone treatment resulted in a decrease in ovarian weight and the ovarian index in the PCOS mice (Supplementary Fig. S2a–c). Hormone levels also returned to normal following progesterone treatment, including testosterone and LH (Supplementary Fig. S2d and e). Histological examination showed that the ovaries of Prog3w group mice possessed fewer advanced follicles than those of PCOS mice, in which follicle development was blocked in the secondary and antral follicle stages (Fig. 2d). The thickness of the endometrium also decreased following progesterone administration in PCOS mice during diestrus (Fig. 2e and f). Moreover, glucose tolerance, but not insulin sensitivity, improved in the Prog3w group compared to the Let group (Supplementary Fig. S2f and g). These findings collectively suggest that progesterone can normalize estrous cyclicity and ameliorate symptoms associated with PCOS.

### Administration of progesterone suppresses ovary function in PCOS mice

**Figure 3:**
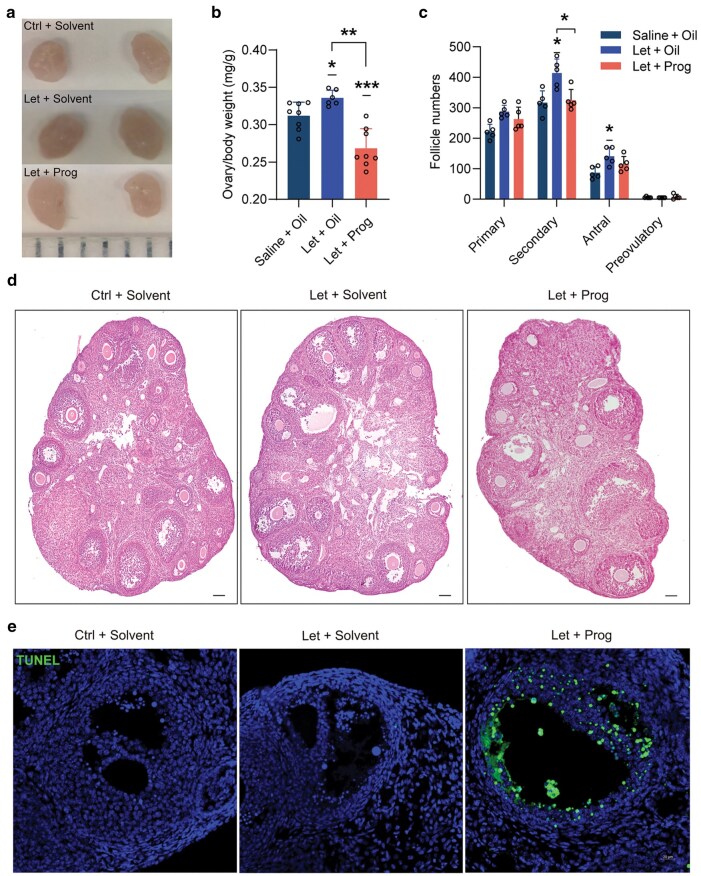
Progesterone treatment suppresses ovary function in PCOS mice. (a) Comparison of ovarian size among control, Let, and Prog groups. (b) Index of ovarian weight of mice in control, Let, and Prog groups (*n *= 6–8). (c) H&E staining of representative ovaries. Scale bar: 200 μm. (d) Quantification of follicles in the ovaries of mice in control, Let group, and Prog groups (*n *= 5). (e) Immunofluorescence staining of ovaries from mice in control, Let group, and Prog groups. Green fluorescence indicates positive TUNEL staining (apoptosis), while blue fluorescence indicates 4′-6-diamidino-2-phenylindole (DAPI) staining (nuclear visualization). Scale bar = 20 μm.

The data presented indicate that progesterone administration in PCOS mice may facilitate subsequent recovery of estrous cyclicity. To further investigate the mechanisms underlying this effect, we collected ovaries at the end of the progesterone treatment period (Prog group). The results showed that both the size and weight of the ovaries were significantly increased in PCOS mice, but reduced following progesterone exposure (Fig. 3a and b). Histological ana­lysis revealed a substantial number of large follicles in the ovaries of PCOS mice; however, progesterone treatment significantly decreased the quantity of secondary and antral follicles in these animals (Fig. 3c and d). In addition, apoptosis analysis indicated that progesterone administration induced apoptosis in the granulosa cells of antral follicles (Fig. 3e). These findings suggest that progesterone inhibits the activities of granulosa cells and causes apoptosis of ovarian granulosa cells. The apoptosis and depletion of large follicles would ensure future development of small follicles when the progesterone level declines after delivery.

### Gestational progesterone downregulates FSHR expression and diminishes the activity of granulosa cells

**Figure 4:**
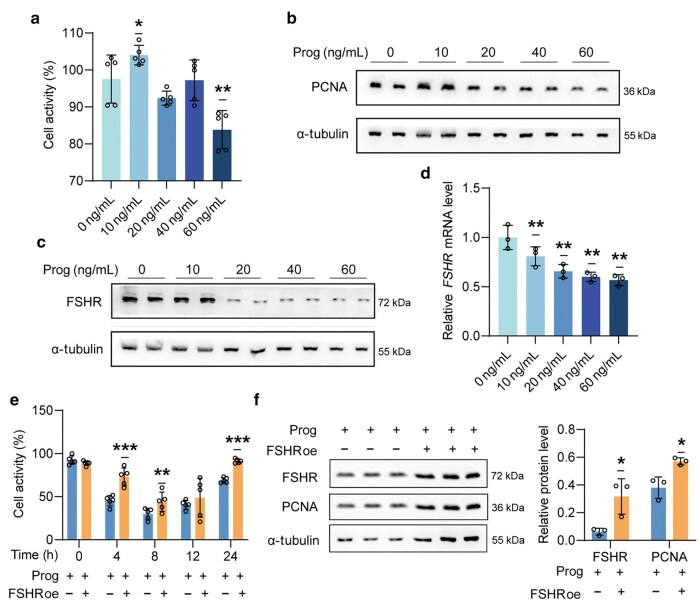
Progesterone treatment blocks granulosa cell activity by decreasing FSHR levels. (a) CCK-8 assay assessing cell activity in the KGN cell line following treatment with various concentrations of progesterone (*n *= 5). (b and c) Western blot analysis of the KGN cell line treated with different concentrations of progesterone. PCNA and FSHR levels were measured, with α-tubulin used as a loading control. (d) The mRNA levels of *FSHR* in the KGN cell line after exposure to different concentrations of progesterone (*n *= 3). (e) CCK-8 assay evaluating cell activity in the KGN cell line after progesterone treatment for varying durations, followed by overexpression of FSHR (*n *= 5). (f) Western blot analysis of the KGN cell line after progesterone treatment and subsequent overexpression of FSHR, along with PCNA measurement. α-tubulin was used as a loading control.

Granulosa cells are capable of converting androgens into estrogens, which play critical roles in follicle development and endometrial growth [13]. We demonstrated that high levels of gestational progesterone suppressed ovarian function by blocking follicle development. Further apoptosis analysis revealed that progesterone administration induced apoptosis in granulosa cells of antral follicles (Fig. 3e). To investigate this effect, we treated human granulosa-like tumor cell line (KGN cells) with progesterone and confirmed that progesterone inhibited both granulosa cell activity (Fig. 4a) and cell proliferation (Fig. 4b). This inhibitory effect on cell activity and proliferation is likely linked to reduced expression levels of FSHR in granulosa cells. Both protein (Fig. 4c) and mRNA (Fig. 4d) levels of FSHR decreased with progesterone administration in a dose-dependent manner. Notably, when we overexpressed FSHR in KGN cells, the inhibitory effects of progesterone on cell activity and proliferation were rescued (Fig. 4e and f). Our results indicate that progesterone suppresses ovarian function by inhibiting the activities of gra­nulosa cells.

### Progesterone regulates FSHR expression through GATA2

**Figure 5:**
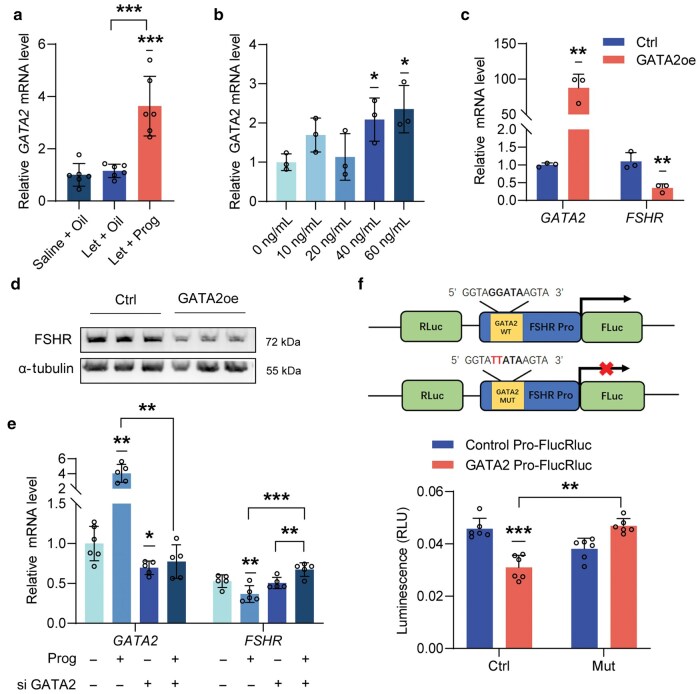
Progesterone decreases FSHR levels through its transcription factor GATA2. (a) Expression levels of GATA2 in the granulosa cells of control, PCOS-like model mice, and progesterone-treated mice (*n *= 6). (b) Expression levels of GATA2 in the KGN cell line after treatment with varying concentrations of progesterone (*n *= 3). (c and d) The mRNA and protein levels of FSHR in control conditions and following GATA2 overexpression (*n *= 3). (e) Expression levels of *GATA2* and *FSHR* after treatment with progesterone and disturbance of GATA2 (*n *= 6). (f) Schematic diagram of the dual-luciferase assays, along with the results of these assays using the promoters of *FSHR* (*n *= 6).

Progesterone signals are conveyed through its nuclear transcription factor, the progesterone receptor (PR) [14]. However, we did not identify any PR binding sites in the promoter region of *FSHR*. After analyzing PR target genes and potential transcription factors within the promoter region of *FSHR*, we focused on GATA binding protein 2 (*GATA2*) to investigate its role in mediating the progesterone regulation of *FSHR* expression. It has been reported that *GATA2* is regulated not only by PR but also by feedback-regulated, progesterone-responsive genes in conjunction with PR [15, 16]. Our findings revealed that progesterone administration increased the expression of *Gata2* in the granulosa cells of PCOS mice (Fig. 5a). The regulation of *GATA2* expression by progesterone was further confirmed in the KGN cells treated with varying concentrations of progesterone (Fig. 5b). In addition, overexpression of *GATA2* inhibited the expression of FSHR at both the mRNA (Fig. 5c) and protein levels (Fig. 5d). These results indicate that progesterone can decrease *FSHR* expression through a GATA2-dependent mechanism (Fig. 5e). To explore the role of *GATA2* on *FSHR* expression, we constructed a luciferase reporter vector driven by the *FSHR* promoter. Overexpression of *GATA2* blocked progesterone-driven transcriptional activity of the *FSHR* promoter; however, this effect was not observed in a luciferase reporter vector where the *GATA2* binding site was mutated (Fig. 5f). In summary, these findings suggest that progesterone reduces *FSHR* expression mediated by *GATA2.*

### Progesterone pretreatment increases estrogen production in granulosa cells following FSH stimulation

**Figure 6:**
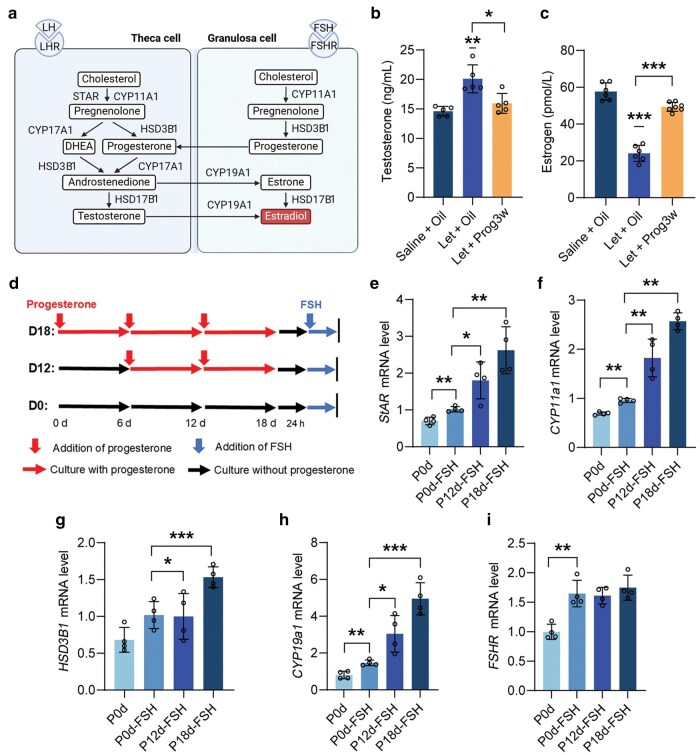
FSH sensitivity increases after progesterone withdrawal. (a) Schematic diagram of hormone synthesis pathway. (b and c) Plasma concentrations of androgen and estrogen levels in mice (*n *= 5–6). (d) Schematic diagram of progesterone treatment and FSH stimulation in KGN cell line. (e–i) Expression levels of *StAR*, *CYP11a1*, *HSD3B1*, *CYP19a1*, and *FSHR* after progesterone treatment and FSH stimulation in KGN cell line (*n* = 4).

In PCOS patients, insufficient secretion of FSH (inhibited by high androgen) and local inhibition of FSH action (FSH resistance) lead to the inability to select a dominant follicle. Granulosa cells are capable of converting androgens into estrogens, which play critical roles in follicle development and endometrial growth [13] (Fig. 6a). The data presented suggest that progesterone administration in PCOS mice may enhance the recovery of estrous cyclicity. To further investigate the underlying mechanisms of this effect, we assessed the function of granulosa cells. We found that circulating levels of both testosterone and estrogen were normalized following progesterone administration in PCOS mice (Fig. 6b and c). This suggests that the estrogen biosynthesis ability of granulosa cells was enhanced following progesterone exposure. We hypothesized that progesterone exposure during pregnancy would increase the estrogen production capacity of granulosa cells in response to FSH stimulation in the subsequent reproductive cycle. To confirm this hypothesis, we pre-treated KGN cells with progesterone for varying durations, specifically for 12 and 18 days (Fig. 6d), with a subsequent rest period of 24 h in progesterone-free media, and then assessed estrogen synthesis capacity followed by a 24-h FSH induction. Gene expression analysis revealed that the expression of steroidogenic enzyme genes, such as steroidogenic acute regulatory protein (*StAR*), cytochrome P450 family 11 subfamily A member 1 (*CYP11a1*), 3beta-hydroxysteroid dehydrogenase type 1 (*HSD3B1*), and cytochrome P450 family 19 subfamily A member 1 (*CYP19a1*), significantly increased with longer progesterone pretreatment (Fig. 6e–h), while there were no significant changes in *FSHR* levels after FSH exposure (Fig. 6i). We isolated primary granulosa cells from progesterone-treated mice and subsequently cultured them *in vitro*. Following FSH treatment, we observed a significant upregulation in the expression of genes involved in the steroidogenesis pathway (Supplementary Fig. S3a–e; see online supplementary material for a color version of this figure). Similarly, we collected primary granulosa cells from PCOS patients and conducted parallel *in vitro* experiments involving progesterone and FSH treatments. The results were consistent, demonstrating a similar upregulation of steroidogenic genes (Supplementary Fig. S3f–j; see online supplementary material for a color version of this figure). The marked elevation in steroidogenic gene expression indicates that progesterone pretreatment can enhance the sensitivity of granulosa cells to FSH stimulation, thereby increasing their capacity for estrogen production.

## Discussion

PCOS is a multifaceted endocrine disorder, with approximately 80% of women affected experiencing infertility related to anovulation along with menstrual dysfunction [17]. PCOS patients ­require hormonal therapy for proper ovarian and uterine function, which is critical for those seeking fertility and a successful pregnancy [18, 19]. Our study found that, following delivery, 60.60% of PCOS patients experienced improved menstrual cycles, with this improvement attributable potentially to elevated progesterone levels during pregnancy. Elevated progesterone may suppress ovarian function by depleting large follicles during pregnancy and enhance estrogen production by augmenting FSH sensitivity in granulosa cells after delivery. These two strategies not only facilitate the restoration of normal ovarian function and ovulation, but also enable the endometrium to respond effectively to hormonal fluctuations through cyclical proliferation and shedding, thereby reestablishing a regular menstrual cycle.

Progesterone serves as a pivotal coordinator of all aspects of ­female reproductive activity during pregnancy. It is produced by the corpus luteum and placenta [20]. The primary physiological roles of progesterone include influencing proliferation and decidualization in the uterus during the menstrual cycle, facilitating implantation, maintaining pregnancy, and promoting mammary gland development [21]. It has been reported that progesterone can suppress follicle development and inhibit antral follicle growth by inhibiting granulosa cell proliferation, differentiation, steroidogenesis, and inflammatory ­responses [22, 23]. However, it has also been reported that progesterone exhibits both stimulatory (1 mg) and inhibitory (1–100 µg) ­effects on follicular development in hypophysectomized hamsters [24]. Therefore, the function of progesterone in the ovary is dose-dependent and species-specific [25, 26]. As the progesterone level reaches a maximum of 202.0 ± 47.0 ng/mL during pregnancy in humans, compared to the normal range of progesterone levels of 6.5–32.2 ng/mL during the luteal phase, we suspected that elevated progesterone and extended treatment could inhibit follicle development and reset ovarian function. Thus, we administered 2 mg progesterone to PCOS mice over a 3-week period, resulting in a physiological level of ­approximately 34.19 ± 2.66 ng/mL, which is comparable to that observed during midterm pregnancy [27]. The treatment resulted in the depletion of larger antral follicles from PCOS ovaries, thereby permitting subsequent follicle development when progesterone levels decline after delivery.

Many patients with PCOS exhibit FSH resistance, which results in a failure to select a dominant follicle. FSH resistance results from increased levels of anti-Mullerian hormone (AMH), which can ­inhibit FSHR expression by reducing cyclic adenosine 3′, 5′-monophosphate (cAMP), the second messenger of FSH [28, 29]. Genetic variations in FSH and its receptor also contribute to the decreased sensitivity to FSH [30]. Pituitary-secreted FSH acts as the master regulator, stimulating aromatase activity and expression, thereby enhancing estrogen production in ovarian granulosa cells. Estrogen can, through feed-forward mechanisms, regulate follicle development and maturation, including granulosa cell survival, proliferation, and steroidogenic differentiation [31]. Our data show that on the one hand, gestational progesterone could suppress ovarian function by inhibiting granulosa cell activity during pregnancy; on the other hand, granulosa cells could retain the long-term progesterone treatment and respond more sensitively to synthesize estrogen when exposed to FSH. This phenomenon resembles what is termed transcription memory [32]. Events of transcriptional memory depend on several mechanisms; the sustained stimulation by progesterone likely induces transcriptional memory through DNA methylation [33].

In addition to progesterone, estrogen and prolactin also experience fluctuations during pregnancy. Our data show that although estrogen treatment restored the estrous cycle of PCOS mice, the ovarian status and hormone levels of the mice were not improved, which suggests that it is not estrogen that is responsible for the improvement of estrous recovery in PCOS mice (Supplementary Fig. S4; see online supplementary material for a color version of this figure). Prolactin levels peak in late pregnancy, reaching levels ten times higher than those observed in nonpregnant women, thereby promoting mammary gland development and preparing for lactation postpartum [34]. Although postpartum prolactin levels generally decrease, each breastfeeding session triggers a significant release of prolactin through neuroendocrine reflexes [35]. Therefore, it is possible that prolactin levels during pregnancy are also responsible for the improvement of PCOS symptoms. We will conduct further investigations to confirm this possibility.

The endometrium is a highly dynamic organ, with its function and cyclicity primarily regulated by ovarian progesterone and estrogen [36]. Estrogen stimulates epithelial proliferation to promote endometrial thickness during the proliferative phase of the menstrual cycle; subsequently, progesterone suppresses estrogen-induced proliferation and facilitates stromal cells to begin the process of decidualization during the secretory phase. If fertilization does not occur, the corpus luteum undergoes degeneration, and the endometrium enters the menstrual phase, resulting in thinning and shedding due to a deficiency of estrogen and progesterone [37]. In PCOS mouse models, the sustained absence of normal hormonal fluctuations leads to abnormal endometrial hyperplasia. Prolonged exposure to progesterone enhances the sensitivity of granulosa cells to FSH, resulting in increased estrogen production. This restoration of cyclic hormonal stimulation acts on the endometrium. Consequently, during diestrus, luteal phase regression results in decreased hormone levels and thinning of the endometrium.

In conclusion, our findings suggest that sustained high levels of progesterone during pregnancy suppress ovarian function by depleting large, arrested follicles. This “ovarian reset” then leads to an enhanced reproductive endocrine capacity after delivery, characterized by increased FSH sensitivity in granulosa cells and improved endometrial function through restored estrogen production, thereby facilitating the recovery of menstrual cycles.

## Limitations of the study

Although we explored the effects of progesterone and estrogen on the estrous cycle in mice, the potential confounding effects of ­other pregnancy-related hormonal changes—such as fluctuations in prolactin and placental hormones—on menstrual recovery have not been fully elucidated in this study. It is necessary to further explore these potential mechanisms. In addition, future prospective, multi-center studies with larger sample sizes and comprehensive hormonal assessments are required to evaluate the long-term persistence of postpartum menstrual improvement. Finally, while progesterone administration showed beneficial effects in mice, the optimal dose, timing, and safety profile of therapeutic progesterone use in non-pregnant PCOS patients require careful investigation prior to clinical application.

## Materials and methods

### Participants

A total of 317 patients with PCOS who had successfully delivered after ART at the Reproductive Center of Nanjing Drum Tower Hospital from January 2015 to December 2016 were enrolled from the previous electronic medical record system. The improvement rate of menstrual rhythm among patients with PCOS was evaluated via telephone follow-up. Data collected included postpartum menstrual cycle rhythm, breastfeeding duration, weight change, and patient-reported menstrual cycle improvement.

### Inclusion and exclusion criteria

#### Inclusion criteria

Patients were diagnosed with PCOS according to Rotterdam ­criteria, revised in 2003. Specifically, PCOS was diagnosed if two of the following three conditions were present: sparse menstruation and/or amenorrhea, biochemical and clinical manifestations of hyperandrogenicity, and polycystic changes in the ovary. Sparse menstruation was defined as a menstrual cycle of more than 35 days. Patients received full-term delivery after *in vitro* ­fertilization/intracytoplasmic sperm injection (IVF/ICSI) fresh ­cycle transfer.

#### Exclusion criteria

Patients who experienced menarche within 3 years, were older than 45 years, or had hyperprolactinemia, hypogonadotropic hypogonadism, premature ovarian failure, congenital adrenal hyperplasia, androgen-secreting tumor, Koch’s syndrome, uterine malformation, chromosomal abnormality, a history of elevated androgen levels, ovarian cyst, or ovarian tumor were excluded from this study.

### Mouse models

All C57BL/6J mice were group-housed under specific pathogen-free conditions in a temperature-controlled room (21°C–22°C) with a 12-h light/12-h dark cycle and *ad libitum* access to food and water. Mice were randomly assigned to groups at the time of purchase to minimize any potential bias. All animal experiments were approved by the Medical School of Nanjing Medical University, Nanjing, China, and performed according to the recommendations of the National Institutes of Health Guide for the Care and Use of Laboratory Animals.

Letrozole (6 mg/kg body weight dissolved in saline) was given orally to adult mice for 21 days. Control mice received saline for the same duration. During the process and treatment, body weight and estrous cycle of rats were recorded to determine whether the PCOS model was successfully established. Mice in Let group were randomly divided into two groups and injected daily intraperitoneally (i.p.) with 2 mg progesterone (MCE, HY-N0437) dissolved in sesame oil (Prog group) or sesame oil alone (control group) for 21 days. E2 (MCE, HY-B0141) was dissolved in saline and injected daily i.p. for 21 days.

### LH, T, and E2 ELISA assays

Plasma LH, T, and E2 levels were analyzed using commercial ELISA kits (Enzyme-linked Biotechnology, ml063366; ml001948; ml001962) according to the manufacturers’ instructions. The assay sensitivity is 0.1 U/L at the 2 standard deviation confidence limit. Intra-assay coefficient of variation for testosterone was 10% and inter-assay ­coefficient of variation was 15%. Totally, 20 uL of plasma was used for the testosterone assay, run in duplicate. As hormone levels can vary significantly due to handling, sampling techniques, and time of day, blood samples were taken from the eyes during diestrus.

### Assessment of estrous cyclicity

During the last 21 days of the experimental period, vaginal smears were obtained daily from all mice between 08:00 and 09:00. Smears were collected via a swab using saline. Samples were mounted and stained with methylene blue. The stage of the ­estrous cycle (i.e., proestrus, estrus, metestrus, and diestrus) was determined and classified by the relative proportion of leukocytes, epithelial cells, and cornified cells by light microscopy.

### Follicle counts

Ovaries were collected and fixed in 4% PBS-buffered parafor­maldehyde, dehydrated in ethanol, embedded in paraffin, serially sectioned at a thickness of 5 μm, and then stained with hemato­xylin and eosin (H&E). All follicles with a visible nucleus were counted in every second section, and analysis was performed on every section. Follicle classification was determined as follows: oocytes surrounded by a single layer of flattened or cuboidal granulosa cells were defined as primordial and primary follicles; oocytes surrounded by more than one layer of cuboidal granulosa cells with no visible antrum were determined to be secondary follicles. Antral follicles possessed a clearly defined antral space and a ­cumulus granulosa cell layer. Corpora lutea were filled with lutein cells. Follicles were considered atretic if they contained either a degenerating oocyte, disorganized granulosa cells, pyknotic nuclei, shrunken granulosa cells, or apoptotic bodies. The results were reported as the number of follicles counted per ovary.

### Glucose tolerance test (GTT) and insulin tolerance test (ITT)

To determine glucose tolerance, mice were injected i.p. with glucose (2 mg/g body weight; Sigma) after 16-h fasting. To test insulin ­sensitivity, mice were given an intraperitoneal injection of insulin (0.7 units/kg body weight; Sigma) after 4-h fasting. Blood glucose levels were determined at the indicated time points (0, 15, 30, 60, 90, and 120 min) from tail blood using the Accu-Check Glucometer (Roche).

### Terminal deoxynucleotidyl transferase-mediated dUTP nick end-labeling (TUNEL) staining

The paraplast sections were mounted on poly-lysine-coated glass slides. After deparaffinization and rehydration, the slides were heated in a microwave oven (3 min in 10 mmol/L citrate buffer, pH 6.0) and rinsed in PBS. The cell death detection kit TUNEL (Promega, G3250) was used, and the protocol of the TUNEL manufacturer was followed. All sections were examined under a confocal microscope (ZEISS, LSM800). The TUNEL assay was repeated two times for each sample with similar results.

### Western blot analysis

For cell and ovary tissues, total protein was extracted from frozen tissues using RIPA lysis buffer containing a protease/phosphatase inhibitor cocktail. The protein concentration in each sample was determined using a BCA Protein Assay (Bio-Rad, Laboratories, Richmond, CA). Equal amounts of protein (20 μg) were separated by 8%−12% SDS-PAGE. The resolved proteins were transferred to polyvinylidene fluoride membranes (Invitrogen) by standard methods to compare the expression of corresponding proteins between different genotypes or treatments. The immunoblots were blocked with 5% milk solution and incubated overnight at 4°C with primary antibodies against FSHR (1:1000, Proteintech, 22665-1-AP), proliferating cell nuclear antigen (PCNA, 1:1000, CST, #13110), and α-tubulin (1:1000, Proteintech, 80762-1-RR), as well as goat anti-rabbit or anti-mouse secondary antibodies (1:5000, Proteintech, SA00001-2; SA00001-1). Immunodetection was carried out using an enhanced chemiluminescence kit (Tanon, 180-5001).

### RNA extraction and reverse transcription quantitative PCR (RT-qPCR)

Ovaries were harvested from mice and snap-frozen in liquid nitrogen. Frozen tissues were homogenized in 1 mL TRIzol (Invitrogen) with a tissue homogenizer. Total RNA was isolated as described in the manufacturer’s instructions and then subjected to reverse transcription with PrimeScript™ RT Master Mix (Takara). The steady-state levels of mRNA in ovaries and cells were determined by SYBR Green-based quantitative real-time PCR analysis. The relative changes in the levels of transcripts between the control and experimental groups were analyzed using the 2^−ΔΔCT^ analysis method, in which 18S rRNA was used as an internal control.

### CCK-8 assay

The KGN cell line was cultured in 96-well plates and analyzed using the Cell Counting Kit-8 (Vazyme, A311) in each experimental group. According to the manufacturer’s instructions, cells were incubated with the CCK-8 solution for 3 h. Optical density was measured at 450 nm with a multifunctional enzyme marking instrument (SPARK, BioTek, USA). In the 96-well plate, the corresponding culture medium with CCK-8 solution (without cells) were used as the blank control, and cells treated with culture medium only (without progesterone treatment) served as negative control.

### Dual-luciferase transient expression assay

The double reporter vector included a native LUC (firefly lucife­rase) and an internal control REN (Renilla luciferase) driven by the *FSHR* promoter, which was modified based on the pGL3.1 reporter vector. For the assay of the binding activity of GATA2 to the promoters of *FSHR*, *GATA2* was cloned into the M35 vector. All primers used for generating constructs for the transient expression assay are listed in Supplementary Table S1. The constructed reporter and effector plasmids were transiently transfected into the 293 cell line. A dual-luciferase assay kit (Vazyme, DL101) was used to analyze transient expression. Absolute LUC/REN was measured with a multifunctional enzyme marking instrument (SPARK, BioTek, USA) according to the manufacturer’s instructions.

### Isolation and culture of primary granulosa cells

Mice in the estrus phase were euthanized by cervical dislocation. The ovaries were removed under sterile conditions and placed in sterile saline. Under a stereomicroscope, the ovaries were cleaned of fat and capsules, washed three times with sterile saline, and then incubated with DMEM. The ovaries were incubated in culture medium at 37°C for 15 min. Follicles were punctured with a 1-mL syringe needle to release granulosa cells. Hyaluronidase (1 g/L) was added, and the mixture was gently pipetted for 1 min, followed by a 20-min digestion. The cell suspension was filtered through a 200-mesh strainer, and centrifuged at 1500 rpm for 5 min. The supernatant was discarded. The pellet was resuspended in 2 mL PBS, centrifuged again, and resuspended in 2 mL of 10% FBS culture medium. Cells were counted and adjusted to 1 × 10^5^ cells per well in a six-well plate. Cultures were maintained at 37°C in a 5% CO_2_ incubator, with the first medium change performed after 24 h and subsequent changes performed every 2–3 days.

### Statistical analysis

All data are presented as mean ± SEM and were analyzed using an unpaired two-tailed Student’s *t*-test between two groups. Multiple groups were tested by one-way ANOVA with Dunnett’s *post-hoc* test or two-way ANOVA followed by Bonferroni’s *post-hoc* test. *P *< 0.05 was considered to be statistically significant. ^*^ ­denotes *P *< 0.05; ^**^ denotes *P *< 0.01; ^***^ denotes *P *< 0.001. All statistical results were performed using GraphPad Prism 9 (GraphPad Software, Inc.).

## Supplementary Material

loag004_Supplementary_Data

## Data Availability

Study protocol and all data collected for the study, including raw data and data analysis, will be made available to others upon request. All data will be available upon publication of the manuscript, by contacting the corresponding author.
