# Supplementary material for: Gestational progesterone restores menstrual cycle in PCOS patients via enhancing ovary estrogen production
Source: Life Metab. 2026 Feb 2;5(3):loag004. doi: 10.1093/lifemeta/loag004 (PMC13128262; doi:10.1093/lifemeta/loag004)
Supplement: loag004_Supplementary_Data [file loag004_supplementary_data.docx]

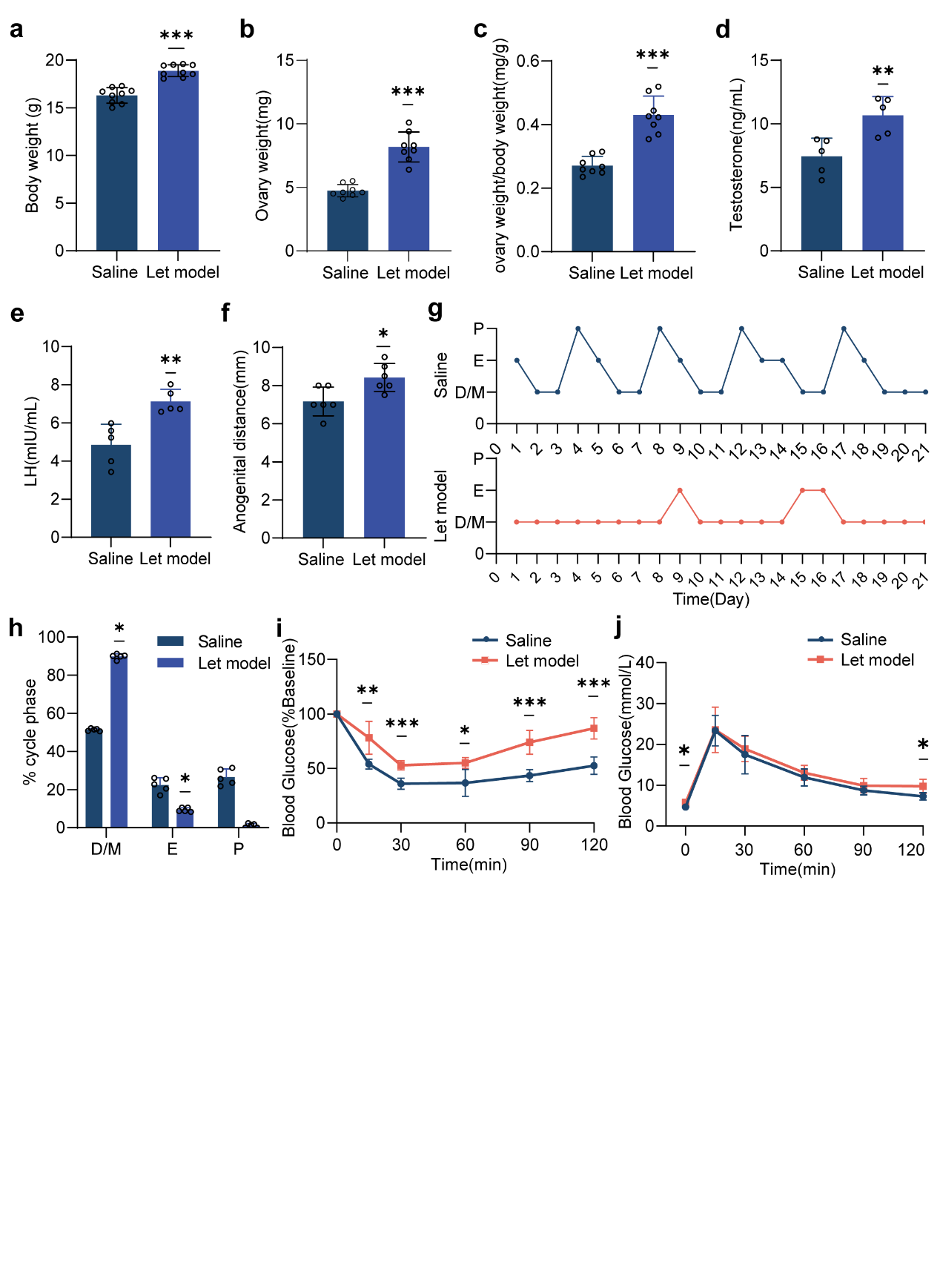


**Supplementary Figure S1** Generation of PCOS-like mouse model. (a−c) Body weight, ovary weight, and ovary index of the control and Let modeling mice (*n* = 8). (d and e) Plasma androgen concentration and LH levels in control and Let modeling mice (*n* = 5). (f) The anogenital distance of the control and Let modeling mice (*n* = 6). (g) Representative estrous cyclicity of control and Let modeling mice over 21 consecutive days. M/D: metestrus/diestrus phase, P, proestrus; E, estrus. (h) Quantitative analysis of estrous cyclicity in control and Let modeling mice, with a scatterplot representing the percentage (%) of time spent in each estrous cycle (*n* = 5). (i and j) Oral glucose tolerance test (GTT) after 14 h of fasting and insulin tolerance test (ITT) after 4 h of fasting in control and Let modeling mice (*n* = 6).


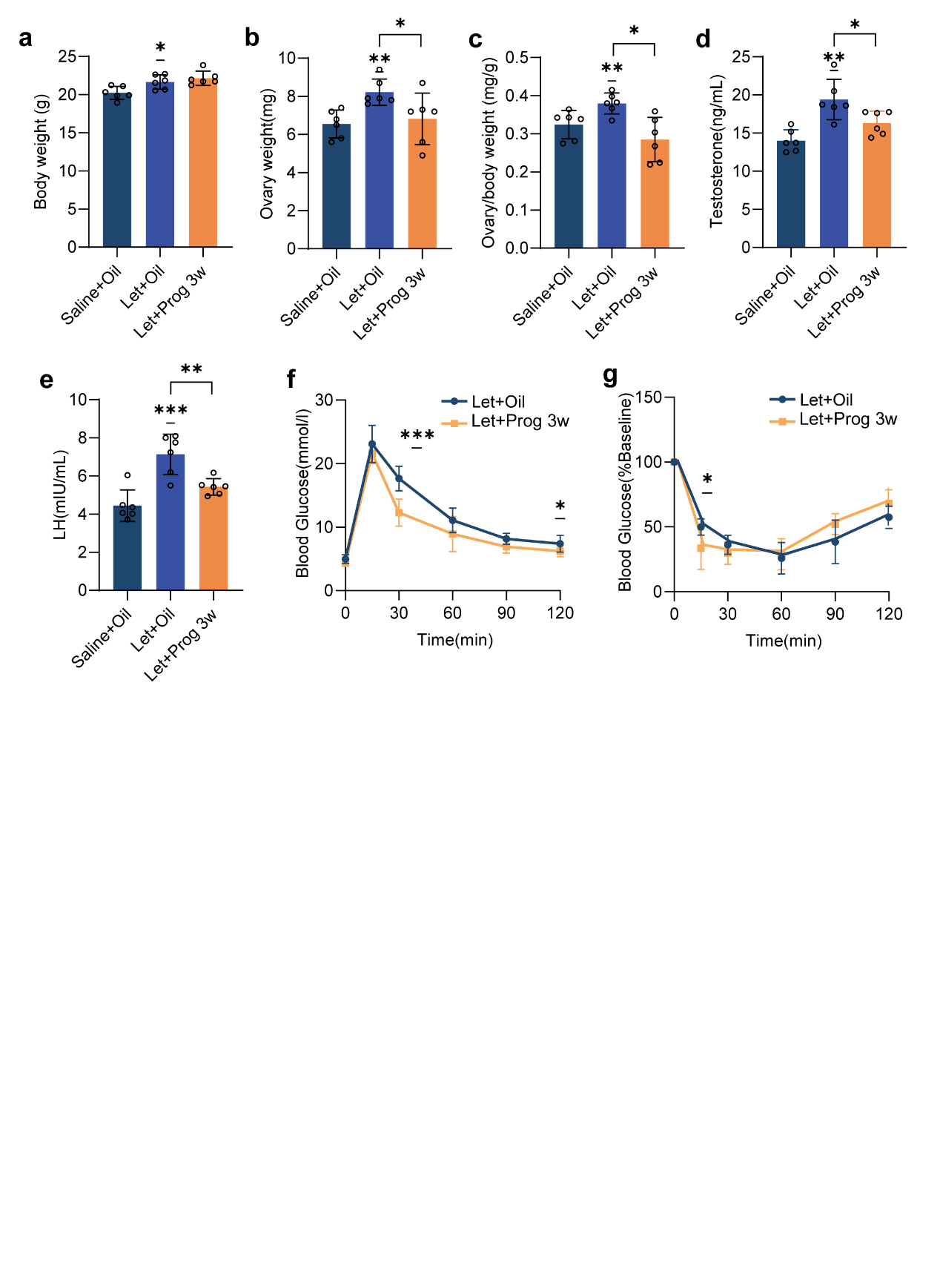


**Supplementary Figure S2** Progesterone treatment improves endocrine reproductive phenotypes in a PCOS-like mouse model. (a−c) Body weight, ovary weight, and ovary index of the Let modeling mice and progesterone-treated mice (*n* = 6). (d and e) Plasma androgen concentration and LH levels in Let modeling mice and progesterone-treated mice (*n* = 6). (f and g) Oral GTT after 14 h of fasting and ITT after 4 h of fasting in Let modeling mice and progesterone treatment mice (*n* = 6).


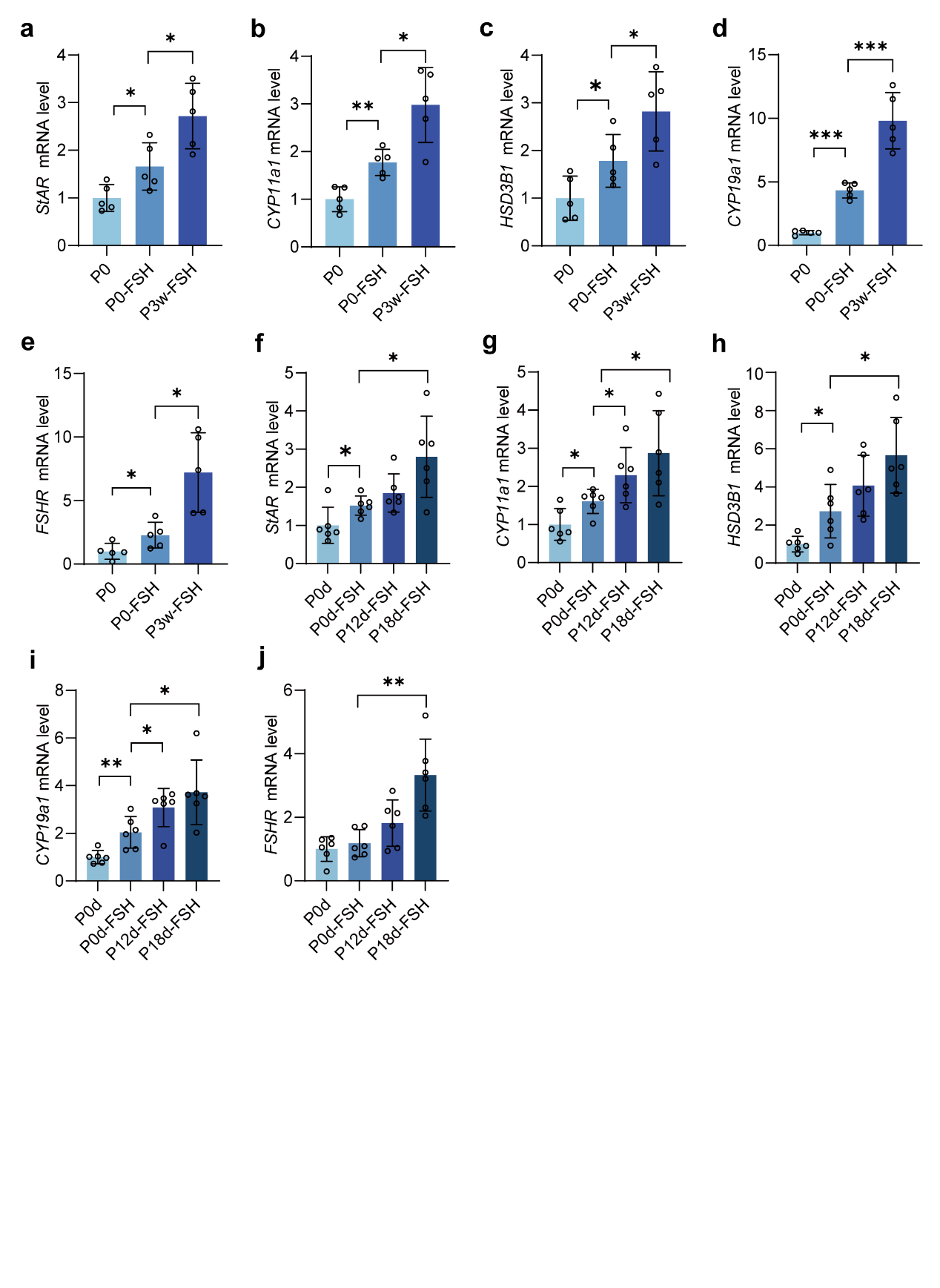


**Supplementary Figure S3** FSH sensitivity increases after progesterone withdraw in primary granulosa cells. (a−e) Expression levels of *StAR*, *CYP11a1*, *HSD3B1*, *CYP19a1*, and *FSHR* after progesterone treatment and FSH stimulation in primary granulosa cells isolated from PCOS model mice (*n* = 5). (f−j) Expression levels of *StAR*, *CYP11a1*, *HSD3B1*, *CYP19a1*, and *FSHR* after progesterone treatment and FSH stimulation in primary granulosa cells isolated from PCOS patients (*n* = 6).


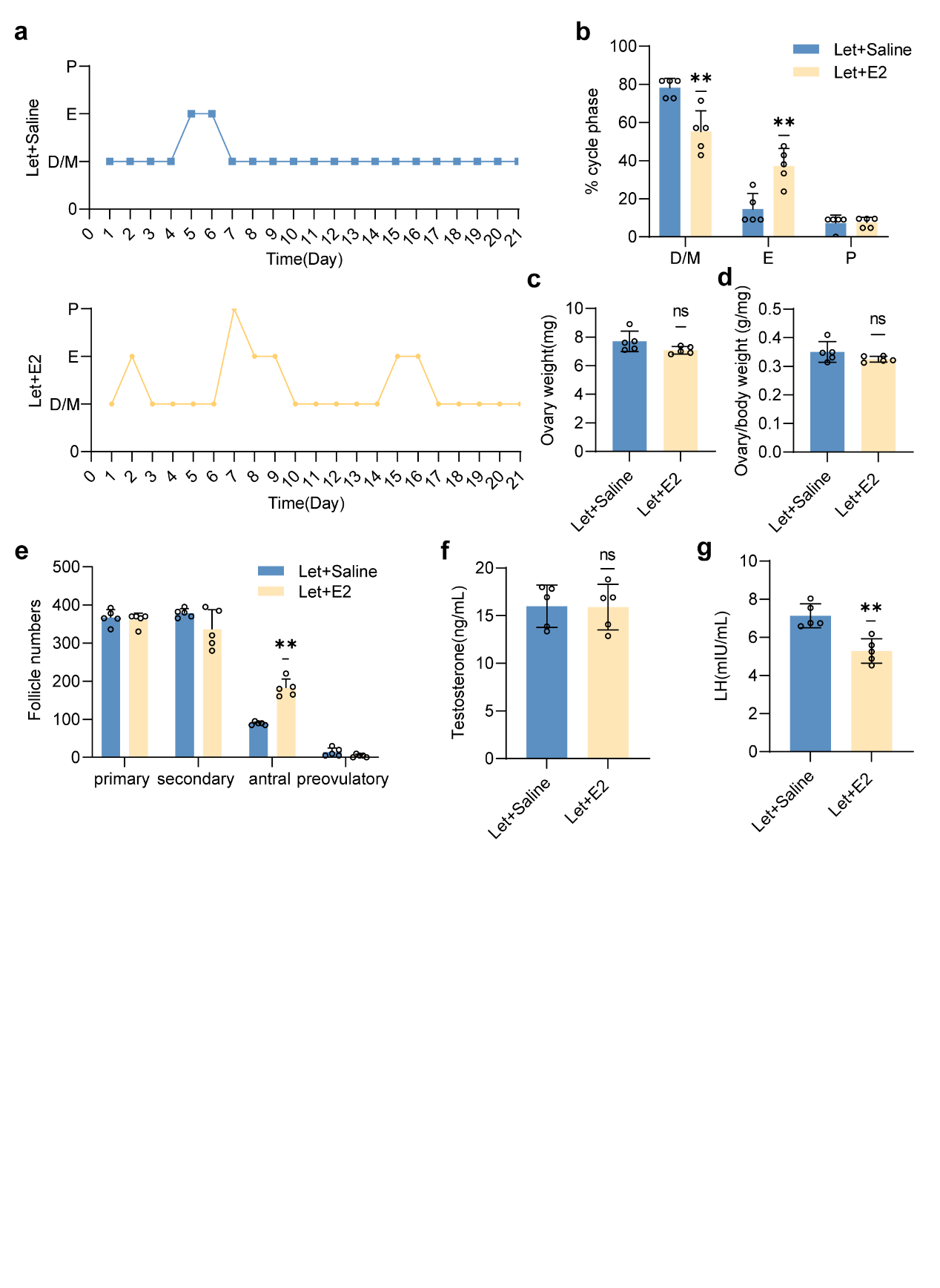


**Supplementary Figure S4** E2 treatment does not change the endocrine reproductive phenotypes of PCOS-like modeling mice. (a) Representative estrous cyclicity of control and E2-treated mice over 21 consecutive days. (b) Quantitative analysis of estrous cyclicity in control and E2-treated mice, with a scatterplot representing the percentage (%) of time spent in each estrous cycle (*n* = 5). (c and d) Ovary weight and ovary index of control and E2-treated mice (*n* = 5). (e) Quantification of follicles in the ovaries of control and E2-treated mice (*n* = 5). (f and g) Plasma testosterone concentration and LH levels in control and E2-treated mice (*n* = 5).

**Supplementary Table S1** List of primer sequences used for real-time PCR analysis.

| Primer | Sequence (5'→3') |
| --- | --- |
| 36B4-F | GAAACTGCTGCCTCACATCCG |
| 36B4-R | GCTGGCACAGTGACCTCACACG |
| hFSHR-F | GCGGAACCCCAACATCGTGTC |
| hFSHR-R | TGAAGAAATCTCTGCGAAAGT |
| hCYP19a1-F | TGGAAATGCTGAACCCGATAC |
| hCYP19a1-R | AATTCCCATGCAGTAGCCAGG |
| hStAR-F | GGGAGTGGAACCCCAATGTC |
| hStAR-R | CCAGCTCGTGAGTAATGAATGT |
| hCYP11a1-F | GCTTTGCCTTTGAGTCCATCA |
| hCYP11a1-R | CTCGGGGTTCACTACTTCCTC |
| hHSD3B1-F | CGGCTAACGGGTGGAATCTG |
| hHSD3B1-R | CCCCATAGATATACATGGGTCGTAAG |
| mFSHR-F | CCTTGCTCCTGGTCTCCTTG |
| mFSHR-R | CTCGGTCACCTTGCTATCTTG |
| mGATA2-F | CACCCCGCCGTATTGAATG |
| mGATA2-R | CCTGCGAGTCGAGATGGTTG |
| mCYP19a1-F | TGTCCAGTGCTCTCACCTATCA |
| mCYP19a1-R | TCCAGACTCGCATGAATTCTCC |
| mStAR-F | TTGGGCATACTCAACAACCA |
| mStAR-R | ATGACACCGCTTTGCTCAG |
| mCYP11a1-F | AGATCCCTTCTCCTGGTGACAATG |
| mCYP11a1-R | TCATACGAATGTCCATCAGCACC |
| mHSD3B1-F | CTGAGGTGGAGGCACTTGTG |
| mHSD3B1-R | TTCCAGAGGCTCTTCTTCGTG |
